# Oncogenic roles of the lncRNA LINC00460 in human cancers

**DOI:** 10.1186/s12935-022-02655-2

**Published:** 2022-07-29

**Authors:** Min Su, Jinming Tang, Desong Yang, Zhining Wu, Qianjin Liao, Hui Wang, Yuhang Xiao, Wenxiang Wang

**Affiliations:** 1 Thoracic Surgery Department 2, The Affiliated Cancer Hospital of Xiangya School of Medicine, Hunan Cancer Hospital, Central South University, Changsha, Hunan 410013 People’s Republic of China; 2 Hunan Clinical Medical Research Center of Accurate Diagnosis and Treatment for Esophageal Carcinoma, The Affiliated Cancer Hospital of Xiangya School of Medicine, Hunan Cancer Hospital, Central South University, Changsha, Hunan 410013 People’s Republic of China; 3 Hunan Key Laboratory of Cancer Metabolism, Hunan Cancer Hospital and The Affiliated Cancer Hospital of Xiangya School of Medicine, Central South University, Changsha, Hunan 410013 People’s Republic of China; 4 Hunan Key Laboratory of Translational Radiation Oncology, The Affiliated Cancer Hospital of Xiangya School of Medicine, Hunan Cancer Hospital, Central South University, Hunan 410013 Changsha, People’s Republic of China; 5 Department of Pharmacy, Xiangya Hospital of Xiangya School of Medicine, Central South University, Changsha, Hunan 410001 People’s Republic of China

**Keywords:** Long noncoding RNA, LINC00460, Human cancer, Oncogenic function, Functional role, Molecular mechanism

## Abstract

Long noncoding RNAs (lncRNAs) represent an important group of endogenous RNAs with limit protein-encoding capability, with a length of more than 200 nucleotides. Emerging evidence have demonstrated that lncRNAs are greatly involved in multiple cancers by playing critical roles in tumor initiation and progression. Long intergenic non-protein coding RNA 460 (LINC00460), a novel cancer-related lncRNA, exhibits abnormal expression and oncogenic function in multiple cancers, and positively correlates with poor clinical characteristics of cancer patients. LINC00460 has also been shown to be a promising biomarker for diagnosis as well as prognostic evaluation in cancer patients. In this review, we briefly summarized recent knowledge on the expression, functional roles, molecular mechanisms, and diagnostic and prognostic values of LINC00460 in human malignancies.

## Introduction

Deep sequencing of mammalian transcriptomes has revealed that approximately 98% of RNA sequences are noncoding RNAs (ncRNAs) that comprise two groups based on length, including small (< 200 nucleotides) and long (lncRNAs; >200 nucleotides) ncRNAs [1, 2]. Over the past two decades, an increasing number of studies have assessed lncRNAs because of their potential involvement in many pathologies, including malignancies [3]. LncRNAs contribute to multiple biological functions in cancer, ranging from cell proliferation, invasion, stemness, angiogenesis, to chemotherapy resistance [4, 5]. LncRNAs were demonstrated to mediate diverse molecular cellular events such as genetic transcription, nuclear compartment formation, alternative splicing and epigenetic modification [6, 7]. For example, lncRNA metastasis-associated lung adenocarcinoma transcript1 (MALAT1) has high expression in non-small cell lung cancer (NSCLC), and promotes proliferation progression of NSCLC cells through stabilizing FOXP3 by inhibited its ubiquitination induced by STUB1 [8]. Glucose transporter 1 (GLUT1) associated lncRNA (GAL) was upregulated in colorectal cancer liver metastasis (CRLM) tissues and associated with the overall survival (OS) rates of CRLM patients. GAL promoted colorectal cancer cell migration and invasion. GAL served as an oncogene through interacting with the GLUT1 protein to increase GLUT1 SUMOylation, inhibiting the effect of the ubiquitin-proteasome system on the GLUT1 protein [9].

Recent evidence reveals the long intergenic non-protein coding RNA 460 (LINC00460, NR_034119) plays a critical role in tumor progression [10]. LINC00460 (on chromosome 13q33.2), is a novel cancer-related lncRNA with a transcript length of 935 nucleotides that contains 3 exons [11, 12]. In the present review, we summarize current research on the expression, functions, underlying mechanism and clinical significance of LINC00460 in human malignancies. Moreover, these provide support for the potential of LINC00460 as a novel biomarker and as a therapeutic target for cancers.

## LINC00460 expression in malignancies

LINC00460 is generally upregulated in multiple tumor cells in comparison to that in control cells (Table 1), including bladder [13, 14], breast [15], cervical [16, 17], colon [18, 19], colorectal [10, 20–24], esophageal [25], gastric [26, 27], ovarian [28], lung [11, 29–31], pancreatic [32] and papillary thyroid cancers [33–35], as well as acute myeloid leukemia (AML) [36], glioma [37], head and neck squamous cell carcinoma (HNSCC) [38–42], hepatocellular carcinoma [43–45], laryngeal squamous cell carcinoma [46], meningioma [47], nasopharyngeal carcinoma [48] and osteosarcoma [49]. In addition, LINC00460 is also overexpressed in these tumor tissues compared with adjacent normal tissues. Its expression level is significantly associated with several clinical characteristics, including tumor size [10, 15, 32, 44, 49], tumor differentiation [25, 41, 42, 44, 45], lymph node metastasis [10, 13, 22, 25, 27, 34, 39, 41, 45], and TNM stage [10, 25, 27, 33, 34, 42, 44, 45].

**Table 1:** Expression and functional characterization of LINC00460 in cancers

Cancer type	Expression in tissue	Sample size	Expression in cancer cells	Cancer cell lines	Relative normal cell lines	Functional role	Refs.
Acute myeloid leukemia	Up	80	Up	THP1, KG1, ME1, HL60	HS5	Proliferation, apoptosis, cell cycle	[36]
Bladder cancer	Up	43	Up	T-24, 5637,SW780, RT-112	SV-HUC-1	Proliferation, migration, invasion	[13]
	–	–	Up	5637, T24	SV-HUC-1	Proliferation, migration	[14]
Breast cancer	Up	42	Up	MCF-7, BT-474, MDA-MB-231, BT-549	MCF-10 A	Proliferation, migration, invasion	[15]
Cervical cancer	Up	20	Up	HeLa, CaSki	–	Proliferation, invasion, cell cycle	[16]
	Up	30	–	SiHa, C-33 A, HeLa, CaSki	–	Proliferation, apoptosis, cell cycle	[17]
Colon cancer	–	–	Up	HT-29, HCT-116, SW480, LOVO	NCM-460	Proliferation, invasion, EMT	[19]
	Up	36	Up	H460, A549, SK-MES-1, and H1299)	NHBE	Invasion, chemoresistance	[18]
Colorectal cancer	Up	60	Up	HCT116, SW480, HT-29, Lovo	HcoEpiC	Proliferation, apoptosis	[10]
	Up	92	Up	SW620, HCT116, CX-1, HT29	NCM460	proliferation, cell cycle	[24]
	Up	74	Up	HT29, HCT116, SW480, and LOVO	NCM460	Proliferation, migration, invasion, apoptosis	[21]
	Up	62	Up	HCT-15, HCT-116, SW480, SW620, RKO, LoVo, HT-29	CCD841CoN	Proliferation, migration, invasion, EMT	[60]
	Up	74	Up	HT29, HCT116, SW480, LOVO	NCM460	Pigration, invasion	[22]
	Up	498	Up	SW480, SW620, HCT116, DLD1, LOVO, HT29	FHC	Proliferation, migration, invasion	[20]
	Up	40	Up	HCT116, HT–29	FHC	Migration, invasion	[23]
	Up	21	–	–	–	Chemoresistance	[64]
Epithelial ovarian cancer	Up	98	Up	SKOV3, A2780, OVCAR, HO–8910	HOSEpiC	–	[28]
Esophageal cancer	–	–	Up	EC1, EC9706, KYSE70, TE1, TE13	Het-1 A	Migration, invasion, EMT	[61]
	Up	65	Up	EC109, KYSE150, KYSE450	Het-1 A	Proliferation, apoptosis	[25]
Gastric cancer	Up	60	Up	MGC803, BGC823 and SGC7901)	GSE1	Proliferation, migration, invasion, cell cycle	[53]
	Up	80	Up	(BGC823, AGS, SGC7901, and MGC803	GES1	Proliferation, apoptosis, cell cycle	[26]
	Up	90	Up	BGC-823, SGC-7901, MKN-28, MKN-45	GES-1	Proliferation, invasion, cell cycle	[27]
Glioma	Up	42	Up	U87, U251, LN229, A172	NHA	Proliferation, migration, invasion, apoptosis	[37]
Head and neck squamous cell carcinoma	Up	15	Up	CAL-27, WSU-HN4, WSUHN6	HOEC	Proliferation, migration,EMT, apoptosis	[38]
	Up	60	Up	HSC3, Fadu, SAS	HACA	Proliferation, migration, invasion, EMT	[39]
	Up	123	Up	WSU–HN4, WSUHN6, WSU-HN30, SCC-4, SCC-9, SCC-25 and CAL-27	Normal oral epithelial cells	Proliferation, migration, invasion, EMT	[41]
	Up	54	–	PCI-13, FaDu, SCC-15, UM-SCC-10 A	–	Apoptosis, autophagy, cell cycle	[42]
Hepatocellular carcinoma	Up	60	Up	SNU423, Hep3B, HuH7, SK-Hep-1	HS-5	Proliferation, migration, invasion, apoptosis	[43]
	Up	60	Up	HepG2, Hep3B, SNU-449, THLE-3, HCCLM3,	Huh-7, LO2	Proliferation, migration, invasion, apoptosis	[44]
	Up	50	Up	HepG2, Huh7, SMMC7721, BEL-7402,HCCLM3, SK-HEP-1	LO2	Proliferation, migration, invasion, cell cycle	[45]
Laryngeal squamous cell carcinoma	Up	68	–	–	–	–	[46]
Lung cancer	Up	50	Up	H157, 95D, SPC-A-1, A549, SK-LU-1, Calu-3, HCC-78, H1299, H1975	16HBE	Proliferation	[11]
	Up	52	Up	A549, H226, H1915, SPCA–1, PC–9	16HBE	Proliferation, migration, invasion, EMT	[29]
	Up	36	Up	H460, A549, SK-MES-1, and H1299	NHBE	Proliferation, invasion, chemoresistance	[30]
	Up	8	Up	A549, H1299, H1975, H460, PC9, SPC-A1	Beas-2B	Migration, invasion, EMT	[31]
Meningioma	Up	33	Up	(IOMM-Lee, CH157-MN)	Ben–Men-1	Proliferation, invasion, apoptosis	[47]
Nasopharyngeal carcinoma	Up	50	Up	SUNE-1, CNE-1, HNE-1, CNE-2, C666-1, HONE-1	NP69	Proliferation	[48]
Osteosarcoma	Up	31	Up	Saos-2, HOS, U2OS, MG63	hFOB 1.19	Proliferation, migration, invasion	[49]
Pancreatic cancer	Up	59	–	–	–	Proliferation	[32]
Papillary thyroid cancer	Up	58	Up	5CTPC1, BCPAP, FTC-133, 8505 C	Nthyori 3-1	Proliferation, migration, invasion, EMT	[33]
	Up	48	Up	TPC-1, BCPAP, IHH-4	Nthyori 3-1	Proliferation, migration, invasion	[34]
	–	–	Up	K1, TPC-1	Nthyori 3-1	Proliferation, invasion, apoptosis	[35]

## Regulation of LINC00460 in cancer

The expression of LINC00460 has been reported to be regulated by genetic and epigenetic methods. A study performed by Zhang and colleagues [50] revealed LINC00460 is increased in colorectal cancer HCT116 cells following irradiation at 2 or 4 Gy. The critical region controlling LINC00460 transcription after irradiation was shown to be between − 240 and − 44 bp upstream of the LINC0460 transcription initiating site. In addition, C-jun was identified as a positive regulator of LINC00460 expression post-irradiation.

Nakano et al. [51] demonstrated that cells transfected with active EGFR mutations have elevated LINC00460 amounts. Furthermore, EGFR activation induced by EGF treatment also caused LINC00460 upregulation, the EGFR-induced increase in LINC00460 expression could be significantly attenuated by gefitinib pre-treatment induced EGFR inactivation. These results suggested that overexpression of LINC00460 was associated with the the abnormal activation of EGFR.

In a report by Zhang et al. [18], LINC00460 showed significant hypomethylation in colorectal cancer tissue samples in comparison with adjacent noncancerous tissue specimens, which had a negative correlation with its expression. In addition, treatment with 5-aza-2’-deoxycytidine resulted in LINC00460 overexpression and demethylation in LOVO and SW620 cells, demonstrating that LINC00460 could be activated by DNA methylation.

In another study, Liang et al. [25] revealed elevated acetyl-histone H3 (Lys18 and Lys27) enrichment signals in the LINC00460 promoter. The chromatin immunoprecipitation (CHIP)-qPCR assay indicated CBP (CREB-binding protein) and P300 (histone acetyltransferase) individually and directly interact with the LINC00460 promoter, and CBP/P300 downregulation decreased LINC00460 amounts in ESCC cells. In addition, both CBP and P300 suppression downregulated LINC00460. These results indicated CBP/P300 interaction with the LINC00460 promoter induces LINC00460 transcription via histone H3 acetylation.

## LINC00460 functions in malignancies

**Fig. 1:**
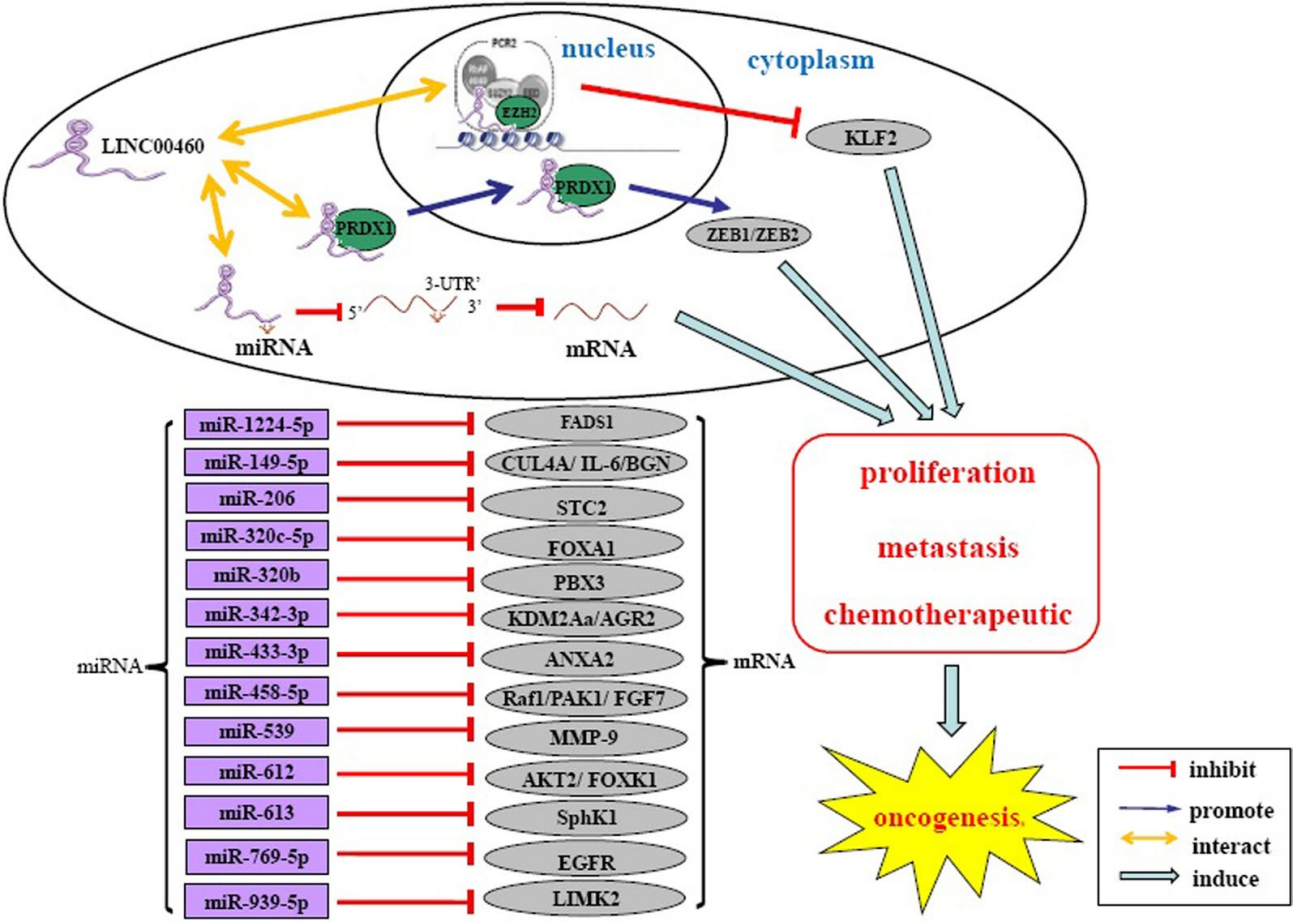
Molecular mechanisms and functional roleunderlying LINC00460 in cancer progression

Studies have proposed that multiple properties contribute to tumor initiation and progression, including sustaining cell growth, resisting cell death, activating invasive and metastatic pathways, and increasing resistance to chemotherapy [52]. Recently, LINC00460 was described for its critical role in controlling oncogenes [11, 13, 15, 19, 21–23, 33, 35, 36, 39, 42–45, 47, 49, 51, 53] and tumor suppressors [10], generally modulating the above mentioned cancer cell features (Fig. 1).

### LINC00460 in cell viability and proliferation

LINC00460 expression is tightly correlated with tumor size in patients with several cancers such as osteosarcoma, and breast, colorectal, liver and pancreatic cancers. such as breast [15], colorectal [10], liver [44], pancreatic [32] cancer, and osteosarcoma [49]. In vitro gain- or loss- of function experiments demonstrated LINC00460 could promote cancer cell proliferation, including acute myeloid leukemia [36], bladder [13, 14], breast [15], cervical [16, 17], colon [19], colorectal [10, 20–24], esophageal [25], gastric [26, 27], ovarian [28], lung, pancreatic [32] and papillary thyroid cancers [33–35], glioma [37], HNSCC [38, 39, 41, 42], hepatocellular carcinoma [43–45], meningioma [47], nasopharyngeal carcinoma [48] and osteosarcoma [49]. Additionally, in vivo tumor xenograft models also demonstrated that silencing LINC00460 reduces tumor volume and lowers tumor weight (Table 2).

**Table 2:** In vivo functional characterization of LINC00460 in cancer

Cancer type	Cancer cell lines	Animal	Role in tumor growth	Refs.
Bladder Cancer	T-24	BALB/c nude mice	Promote	[59]
Breast cancer	MCF-7	Nude mice	Promote	[15]
Cervical cancer	HeLa, CaSki	Nude mice	Promote	[16]
	SiHa	Nude mice	Promote	[17]
Colon cancer	HCT-116, LOVO	BALB/c nude mice	Promote	[19]
Colorectal cancer	HCT116	Nude mice	Promote	[20]
	HCT116, SW480	BALB/c nude mice	Promote	[10]
	HT29	BALB/c nude mice	Promote	[21]
	RKO	BALB/c nude mice	Promote	[60]
Gastric cancer	BGC823	Athymic mice nude mice.	Promote	[26]
	MKN-45	Nude mice	Promote	[27]
Head and neck squamous cell carcinoma	CAL-27	BALB/c nude mice	Promote	[41]
	Fadu	BALB/c nude mice	Promote	[39]
Hepatocellular carcinoma	Hep3B	BALB/c nude mice	Promote	[44]
	HepG2	BALB/c nude mice	Promote	[45]
	HuH7	BALB/c nude mice	Promote	[43]
Lung cancer	A549, SPC-A-1	BALB/c nude mice	Promote	[11]
	A549	Nude mice	Promote	[30]
Nasopharyngeal carcinoma	5-8 F	BALB/c nude mice	Promote	[62]
	CNE-1, SUNE-1	BALB/c nude mice	Promote	[48]
Papillary thyroid cancer	TPC1	BALB/c nude mice	Promote	[33]

Studies have shown that LINC00460 knockdown significantly suppressed cancer cell progression at the G1 phase of the cell cycle. The function of LINC00460 on cell cycle progression might be related to the regulation of LINC00460 on protein proteins relevant to cell cycle, including cyclin D1 [17, 44, 54], CDK2 [26], CDK4/CDK6 [54], CCNG2 [26], CCND1 [53].

### LINC00460 in cell death

Apoptotic, autophagic and necrotic cell deaths are the main mechanisms of cell death [55]. Cell apoptosis is the common process of programmed cell death [56]. LINC00460 has been reported to inhibit apoptosis in various cancers. The anti-apoptotic function of LINC00460 might be related to the regulation of the apoptotic proteins caspase-3 [21, 36], caspase-9 [10], PARP [17], Bcl-2 and Bax [10, 44].

LINC00460 could also regulate autophagy in cancer cells [42]. Knockdown of LINC00460 resulted in increased amounts of autophagosomes in HNSCC cells, along with increased LC3 II/LC3 I ratio and Beclin 1 amounts. Meanwhile, overexpression of LINC00460 restrains autophagy, with reduced number of autophagosomes and decreased LC3 II/LC3 I ratio and Beclin 1 amounts.

### LINC00460 in cancer metastasis

Metastasis is the major cause results in the high mortality rate of diverse types of cancer, and high rates of metastasis are characteristic of advanced malignancies [57, 58]. LINC00460 has been reported to be positively associated with lymph node and distant metastases as well as TNM stage in diverse malignancies, including bladder [59], colorectal [21, 22, 24], esophageal [25], gastric [27], head and neck [39, 41, 42], liver [45] and papillary thyroid [34] cancers, as well as osteosarcoma [49]. In vitro experiments demonstrated LINC00460 could regulate migratory and invasive pathways in malignant cells. A role for LINC00460 in metastasis has also been documented, primarily involving the regulation of epithelial-to-mesenchymal transition (EMT), in which epithelial cells undergo diverse modifications to acquire a mesenchymal phenotype. Several studies have demonstrated that LINC00460 knockdown inhibits EMT development and regulates the expression of proteins relevant to EMT (upregulate E-cadherin, downregulate N-cadherin and vimentin) [19, 22, 29, 38, 39, 41, 50, 54, 60–62].

### LINC00460 in chemotherapeutic or radiation resistance

At present, intrinsic or acquired resistance is the main cause of chemotherapy failure in many cancers [63]. LINC00460 has been demonstrated to be involved in chemoresistance. Zhang et al. [18] investigated the associations of lncRNAs and antitumor drug response, and demonstrated that LINC00460 could distinguish responses to AZD6244 and PD-0325901 in colon cancer samples. Meng and colleagues [64] demonstrated that LINC00460 is upregulated in colorectal cancer cells with oxaliplatin resistance and p53 mutations, compared with parental oxaliplatin-sensitive cells. LINC00460 silencing sensitized oxaliplatin-resistant colorectal cancer cells to this drug via p53 regulation.

The expression of LINC00460 was shown to be elevated in gefitinib-resistant NSCLC and cells [30]. Gain- and loss of function assays showed LINC00460 induces gefitinib resistance by increasing the expression of EGFR and the multidrug-resistance-associated proteins P-gp, MRP1, and BCRP. In another study, Nakano and collaborators [51] demonstrated that LINC00460 amounts are markedly elevated in cancer with wild-type or mutated (exon 19 deletion and L858R) EGFR in comparison with noncancerous tissues. It was also upregulated in NSCLC cells with gefitinib resistance in comparison with gefitinibsensitive cells. EGFR activation, induced by transfection with active EGFR mutations or treatment with EGF, resulted in higher LINC00460 expression, suggesting LINC00460 contributes to resistance against EGFR-TKIs.

Radiation therapy is broadly utilized for treatment of some solid tumors, and recent advances enable direct tumor targeting, without harming adjacent noncancerous tissues [65]. Radiation treatment is mostly hampered by tumor resistance, and decreasing recurrence post-radiotherapy represents an important challenge [66]. LINC00460 was shown to be markedly upregulated following irradiation at 2 or 4 Gy in HCT116 cells [50]. Transient LINC00460 silencing remarkably reduced HCT116 cell proliferation and EMT induced by irradiation. Thus, LINC00460 was considered to mediate the sensitization of HCT116 cells to ionizing radiation.

## Mechanisms underlying LINC00460’s effects in malignancies

Mounting evidence suggests the regulatory mechanisms of lncRNAs include modulating epigenetic alterations, regulating transcription or splicing, interacting with RNA binding proteins, and acting as miRNA sponges [4]. LncRNAs are involved in the regulation of various biological functions in the nucleus and cytoplasm [67]. LINC00460 was shown to be subcellular distributed in both cytoplasm and nucleus, thus playing important modulatory roles at the transcriptional and post-transcriptional levels [10, 11, 25, 48, 60] (Fig. 1). The following sections mainly focus on the molecular mechanisms of LINC00460 in regulating biological functions of malignancies.

### LINC00460 serves as a ceRNA

One important mechanism of lncRNA is function as competing endogenous RNA (ceRNA), through sponging miRNA from target mRNA of the miRNAs and constructing a triple network of lncRNA-miRNA-mRNA. Several studies have shown LINC00460 is primarily expressed in cytoplasm of cells, and thus could act as a ceRNA through interaction with miRNAs, including miR-1224-5p [61], miR-149-5p [10], miR-206 [42], miR-302c-5p [11], miR‑320b [36], miR-342-3p [53], miR-433-3p [19], miR-539 [35], and so on (Table 3). In addition, several assays, such as luciferase reporter assays and RNA immunoprecipitation (RIP) and/or RNA pull-down assays, were performed to identify miRNA-binding sites on LINC00460. Furthermore, functional assays indicated the miRNA and its target mRNA control LINC00460’s effects.

**Table 3:** CeRNA function of LINC00460 in cancer

LINC00460 target miRNA	Validated method	miRNA target gene	Cancer type	Refs.
miR-1224-5p	Luciferase reporter assay	–	Esophageal cancer	[61]
miR-1224-5p	Luciferase reporter assay, RIP	FADS1	osteosarcoma	[49]
miR-149-5p	Luciferase reporter assay, RIP	CUL4A	colorectal cancer	[10]
miR-149-5p	Luciferase reporter assay, RNA pull–down	IL6	nasopharyngeal carcinoma	[48]
miR-149-5p	Luciferase reporter assay	BGN	colorectal cancer	[23]
miR1495p	Luciferase reporter assay, RIP	IL6	Lung adenocarcinoma	[51]
miR-149-5p, miR-150-5p	Luciferase reporter assay, RNA pull–down	p53	Colorectal Cancer	[64]
miR-206	Luciferase reporter assay, RNA pull–down	STC2	Head and neck squamous cell carcinoma	[42]
miR-302c-5p	Luciferase reporter assay, RNA pull–down	FOXA1	Lung adenocarcinoma	[11]
miR30a3p	Luciferase reporter assay, RNA pull–down	–	Nasopharyngeal carcinoma	[62]
miR-320a	Luciferase reporter assay	–	Glioma	[37]
miR320b	Luciferase reporter assay	PBX3	Acute myeloid leukemia	[36]
miR-338-3p	Luciferase reporter assay	–	Epithelial ovarian cancer	[28]
miR-342-3p	Luciferase reporter assay, RIP	KDM2A	Gastric cancer	[53]
miR-342-3p	Luciferase reporter assay, RNA pull–down	AGR2	Hepatocellular carcinoma	[45]
miR-342-3p	Luciferase reporter assay	AGR2	Hepatocellular carcinoma	[43]
miR3613p	Luciferase reporter assay	Gli1	Cervical cancer	[16]
miR-433-3p	Luciferase reporter assay, RNA pull–down	ANXA2	Colon cancer	[19]
miR-4443	Luciferase reporter assay	–	Head and neck squamous cell carcinoma	[38]
miR-485-5p	Luciferase reporter assay	Raf1	Papillary thyroid cancer	[33]
miR-485-5p	Dual luciferase reporter assay, RNA pull–down assay, RIP	PAK1	Hepatocellular carcinoma	[44]
miR-489-5p	Luciferase reporter assay, RNA pull–down	FGF7, AKT	Breast cancer	[15]
miR-5035p	Luciferase reporter assay	AKT2, HMGA2, SHOX2	Cervical cancer	[17]
miR-539	Luciferase reporter assay	MMP–9	Meningioma	[47]
miR-539	Luciferase reporter assay	MMP–9	Papillary thyroid carcinoma	[35]
miR-612	Luciferase reporter assay, RIP	AKT2	Head and neck squamous cell carcinoma	[39]
miR-612	Luciferase reporter assay	FOXK1	Bladder Cancer	[59]
miR-613	Luciferase reporter assay, RIP	SphK1	Colorectal cancer	[21]
miR-939-5p	Luciferase reporter assay, RNA pull–down	LIMK2	Colorectal cancer	[22]

### LINC00460 interacts with RNA binding proteins

LncRNAs has been shown to control gene expression by interacting with RNA binding proteins (RBPs) [68]. RNA pull-down assays and mass spectroscopy are generally performed in sequence for identifying RBPs for lncRNAs. Utilizing this method, Jiang et al. revealed PRDX1 as an RBP that binds LINC00460 [41]. The interaction between PRDX1 and LINC00460 was confirmed by RIP assays. In a further report, Li and co-workers [31] showed hnRNP K is a RBP that binds to LINC00460, and confirmed LINC00460 interacts with hnRNP K by immunoblot.

Apart from mass spectrometry, bioinformatics is also generally carried out for predicting the odds of LINC00460 interacting with RBPs, followed by confirmation by the RIP assay. Using this method, Yang and collaborators [26] showed LINC00460 interacts with EZH2 and LSD1, inducing H3K27 trimethylation and H3K4 demethylation of target gene promoters, thereby suppressing transcription. In addition, LINC00460 interactions with EZH2 and LSD1 were also confirmed by the ChIP assay. In another report, Lian et al. [10] predicted LINC00460 could potentially bind to EZH2, SUZ12, DNMT1, and AGO2, by using bioinformatics analysis. They further confirmed that LINC00460 interacts with EZH2 through RIP assays, and further regulates the expression of KLF2.

## LINC00460 as a cancer biomarker

### LINC00460 as a molecular marker for cancer diagnosis

It is now widely accepted that the early diagnosis is crucial for achieving a lower mortality rate of tumors [69]. The detection and identification of lncRNAs in body fluids, including serum and plasma, may provide a novel tool for early noninvasive diagnosis of cancer [70]. Serum LINC00460 amounts were markedly elevated in 80 AML or CN-AML cases compared with 67 healthy control cases [36]. Receiver operating characteristic (ROC) curve analysis revealed serum LINC00460 amounts provided a clear separation of AML and healthy controls, with an area under the curve (AUC) of 0.8488 (95% CI, 0.7697–0.9279). Additionally, serum LINC00460 reliably differentiated CN-AML cases from healthy control cases (AUC = 0.7591). Serum LINC00460 amounts were also markedly reduced in patients after complete remission. These findings suggested that LINC00460 might be a potential diagnostic biomarker for patients with AML.

However, LINC00460 is expressed in a broad range of cancer types, making it less specific in distinguishing the origin of tumors. Further studies for the expression, sensitivity and stability of LINC00460 in non-invasive body fluids should be further investigated for are required to make LINC00460 an ideal tool for disease diagnosis. In addition, LINC00460 in body fluids are required to investigate for its diagnostic value alongside other specific molecular markers, and further investigations with larger clinical sample sizes are still required.

### LINC00460 serves as a biomarker for cancer prognosis

Recently, aberrant expression of LINC00460 has been considered an independent prognostic factor in diverse cancers. Indeed, elevated LINC00460 amounts were significantly associated with poor OS in bladder cancer [13, 71], cervical cancer [16, 17], colorectal cancer [10, 20–22], esophageal cancer [25, 72], gastric cancer [27], HNSCC [39, 40], liver cancer [45], lung cancer [30, 31, 51], osteosarcoma [49, 54], pancreatic cancer [32] and papillary thyroid carcinoma [33]. In addition, upregulation of LINC00460 was also associated with poor progression free survival (PFS) in AML [36], colorectal cancer [20, 23, 24], gastric cancer [26, 27], glioma [37], hepatocellular carcinoma [45] and lung adenocarcinoma [51] and osteosarcoma [54]. Besides survival data, other clinical features including tumor size, histological grade, differentiation degree, lymph node metastasis and TNM stage, are related to LINC00460 expression, (Table 4).

**Table 4:** Involvement of LINC00460 in cancer prognosis

Cancer type	Prognostic indicator	Associated clinical features	Refs.
Acute myeloid leukemia	OS, PFS	FAB classification, cytogenetics	[36]
Bladder cancer	OS	Tumor stage, lymph nodes metastasis	[59, 71]
Breast cancer	OS	Tumor size, WHO stage	[15]
Cervical cancer	OS	–	[16, 17]
Colon cancer	OS	–	[18]
Colorectal cancer	OS, DFS	Tumor stage, metastasis classification, lymph node metastasis, TNM stage	[10, 20–24]
Esophageal squamous cell carcinoma	OS	TNM stage, lymph node metastasis, differentiation degree	[25]
Gastric cancer	OS, DFS	TNM stage, lymph node metastasis	[26, 27]
Head and neck squamous cell carcinoma	OS	Tumor stage, tumor differentiation, lymph node metastasis, TNM stage,	[39–42]
Hepatocellular carcinoma	OS, PFS	Tumor differentiation degree, TNM stages, lymph node metastasis	[44, 45]
Lung cancer	OS, PFS	–	[30, 31, 51]
Nasopharyngeal carcinoma	OS	–	[48, 62]
Osteosarcoma	OS, DFS	Tumor size, distant metastasis	[49, 54]
Pancreatic cancer	OS	Tumor size	[32]
Papillary thyroid carcinoma	OS	TNM stage, lymph node metastasis	[33, 34]

The prognostic value of LINC00460 was further investigated in combination with other lncRNAs. Cao and colleagues [73] identified an lncRNA trio (LINC00460, KTN1-AS1 and RP5-894A10.6) jointly showing an AUC of 0.68 (95% CI 0.60–0.76, P < 0.0001). In addition, Kaplan-Meier analysis of HNSCC cases, categorized into the high- and low-risk groups according to lncRNA signature-based risk score, revealed marked OS differences between the high- (43.9 months) and low- (25.6 months) risk groups (P = 0.002 in the log-rank test). The findings suggested the three-lncRNA panel-based signature could effectively predict patient survival in HNSCC.

Zhang et al. [74] conducted a lncRNA prognostic model with another lncRNA trio, comprising LINC00460, MIAT and LINC00443, which could independently distinguish kidney renal clear cell carcinoma cases at low- and high-risk of poor OS, with AUCs for 1-, 5- and 10-year OS of 0.723, 0.714 and 0.826, respectively. The model had independent and great prognostic value in these patients.

In another study, Huang et al. [72] identified another lncRNA trio, comprised of RP11-366H4.1.1, LINC00460 and AC093850.2, as an efficient predictive factor of OS and DFS in patients with ESCC. The authors utilized multivariable Cox regression analysis to generate a risk score as (0.882 × AC093850.2)+(1.219 × LINC00460)+(0.921 × RP11-366H4.1.1), whose cutoff was 48.48. Median OS was markedly reduced in high-risk cases compared with the low-risk group in the training set (23.1 months vs. 39.1 months, P < 0.001), the test set (23 months vs. 59 months, P < 0.001) and an independent esophageal squamous cell carcinoma dataset (GSE53624) (22.4 months vs. 60.4 months, P < 0.001). In addition, the three-lncRNA signature could also be used for predicting DFS, with median DFS times of 15.2 and 33.3 months in high- and low-risk cases of the training set, respectively (P < 0.001), versus 16.4 and 50.8 months in the test set, respectively (P < 0.001). The above findings demonstrated the prognostic capability of the three-lncRNA signature to predict survival and recurrence risk.

Therefore, LINC00460 in combination with other lncRNAs or specific biomarkers can function as an independent prognostic indicator in diverse cancer types.

## Conclusion and future perspectives

Numerous studies have confirmed that lncRNAs play critical roles in tumor development and progression in humans. This review mainly discusses research progress of the role, mechanism and clinical value of LINC00460 in a variety of human tumors. LINC00460 has been demonstrated to be upregulated in major types of human malignancies, regulating cellular events such as cell proliferation, apoptosis, migration, invasion, and chemoresistance. Thus, LINC00460 might be a potential candidate for treating diverse cancer types. Mechanistically, LINC00460 might modulate genes via a ceRNA mechanism or by interacting with RBPs. However, how LINC00460 is dysregulated in cancer remains incompletely defined. Regarding clinical application, LINC00460 dysregulation is associated with patient survival in many cancer types, and may also constitute a potent noninvasive molecular marker for diagnosing malignancies, indicating LINC00460 might represent a potential diagnostic and prognostic molecular marker. Overall, the above data indicate upregulation of and an oncogenic role for LINC00460 in human cancer.

However, there is a need for additional basic and clinical experimental results before LINC00460 can be applied in the clinic. Firstly, the actual molecular mechanism and regulatory effect of LINC00460 needs to be further explored. Secondly, many of these findings were established in tissues and cancer cell lines, lacking clinical correlation. Thirdly, although strategies such as lentivirus or plasmid containing siRNA have been used to target LINC00460 in vitro, the in vivo delivery vector for therapeutic lncRNA is still need to be developed. In summary, basic research on LINC00460 has shown encouraging results, it is expected to achieve breakthroughs in diagnosis, prognosis evaluation, and treatment in clinical trials.

## Data Availability

Not applicable.

## Abbreviations

AML: Acute myeloid leukemia
AUC: Area under the curve
ceRNA: Competing endogenous RNA
ChIP: Chromatin immunoprecipitation
CRLM: Colorectal cancer liver metastasis
EMT: Epithelial-to-mesenchymal transition
GAL: GLUT1 associated lncRNA
GLUT1: Glucose transporter 1
HNSCC: Head and neck squamous cell carcinoma
LINC00460: Long intergenic non-protein coding RNA 460
lncRNAs: Long noncoding RNAs
ncRNAs: Noncoding RNAs
MALAT1: Metastasis-associated lung adenocarcinoma transcript1
NSCLC: Non-small cell lung cancer
OS: Overall survival
PFS: Progression free survival
RBP: RNA binding protein
RIP: RNA immunoprecipitation
ROC: Receiver operating characteristic
TNM: Tumor Node Metastasis
